# Neuromuscular adaptations after osseointegration of a bone-anchored prosthesis in a unilateral transfemoral amputee – a case study

**DOI:** 10.1080/07853890.2023.2255206

**Published:** 2023-09-07

**Authors:** Thomas Krauskopf, Torben Lauck, Britta Meyer, Lukas Klein, Marc Mueller, Johanna Kubosch, Georg Herget, Vinzenz von Tscharner, Jennifer Ernst, Thomas Stieglitz, Cristian Pasluosta

**Affiliations:** a Laboratory for Biomedical Microtechnology, Department of Microsystems Engineering, University of Freiburg, Freiburg, Germany; b BrainLinks-BrainTools Center, University of Freiburg, Freiburg, Freiburg, Germany; c Department of Orthopaedics and Trauma Surgery, Medical Center, University of Freiburg, Freiburg, Germany; d Sanitätshaus Pfänder, Freiburg, Germany; e Human Performance Laboratory, University of Calgary, Calgary, Canada; f Department of Trauma Surgery, Hannover Medical School, Hannover, Germany; g Department of Trauma Surgery, Orthopedics and Plastic Surgery, University Medical Center Goettingen, Göttingen, Germany; h Bernstein Center Freiburg, University of Freiburg, Freiburg, Germany

**Keywords:** Amputee, neuromuscular control, osseointegration, balance, bone anchoring

## Abstract

**Purpose:**

Many individuals with a lower limb amputation experience problems with the fitting of the socket of their prosthesis, leading to dissatisfaction or device rejection. Osseointegration (OI)- the implantation of a shaft directly interfacing with the remaining bone- is an alternative for these patients. In this observational study, we investigated how bone anchoring influences neuromuscular parameters during balance control in a patient with a unilateral transfemoral amputation.

**Material and methods:**

Center of pressure (CoP) and electromyography (EMG) signals from muscles controlling the hip and the ankle of the intact leg were recorded during quiet standing six months before and one and a half years after this patient underwent an OI surgery. Results were compared to a control group of nine able-bodied individuals.

**Results:**

Muscle co-activation and EMG intensity decreased after bone anchoring, approaching the levels of able-bodied individuals. Muscle co-activation controlling the ankle decreased in the high-frequency range, and the EMG intensity spectrum decreased in the lower-frequency range for all muscles when vision was allowed. With eyes closed, the ankle extensor muscle showed an increased EMG intensity in the high-frequency range post-surgery. CoP length increased in the mediolateral direction of the amputated leg.

**Conclusions:**

These findings point to shifts in the patient’s neuromuscular profile towards the one of able-bodied individuals.

## Introduction

Prosthetic devices for the lower limb have evolved from being made from simple wood logs to prostheses having microprocessor-controlled knees [1–4]. These technological advances allow lower limb amputees to adjust to different situations such as transitioning from overground walking to stair climbing. While commercially available prostheses restore some mechanical functions of the missing limb, they still provide only limited sensory feedback [5,6] with few available options [7]. Without artificial sensory feedback, amputees usually rely on sensations from the residual limb (RL), the intact leg, and vision [8,9]. This results in neuromuscular problems, such as weight-bearing asymmetries leading to atrophy of the remaining stump muscles, destabilization of the hip [10,11], and increased postural sway while standing on compliant surfaces [12,13]. These neuromuscular problems are usually reflected in altered dynamics of the center of pressure (CoP) adjustments and altered muscle co-activation during quiet standing [14,15]. The CoP represents the average position of the vertical ground reaction forces resulting from the forces exerted by the feet over the ground [16–18]. A pressure platform is commonly used to measure the changes in the CoP over time, with complex variability revealing the individual’s ability to counteract external and internal perturbances [18–20].

An important aspect of lower limb prostheses is their mechanical anchoring to the RL. Conventional prostheses are attached to the stump *via* a socket that surrounds the RL. These sockets may cause skin problems, increased sweating, reduced air circulation, and eventually result in largely poor fit and discomfort [21,22]. These problems further reduce the patients’ quality of life and increase device rejection and exclusion from social activities [23–25].

Bone-anchored prostheses present a promising alternative to conventional socket attachment [26,27]. In this type of anchoring, an implant is inserted into and osseointegrated by the remaining bone, replacing the socket of the prostheses. The patient’s quality of life is significantly enhanced by bone-anchored prostheses because they reduce discomfort while sitting, eliminate skin irritations, and restore the hip range of motion [28]. Amputees also gain an enhanced feeling of the weight of the prosthesis, and they can even sense the ground surface while walking [29,30]. This extended sensory feedback – called osseoperception – leads to an improved and altered muscle activation on the amputated side, indicating the influence of the osseointegration on the neuromuscular system [31,32].

Recent findings showed that amputees who underwent OI and direct bone anchoring of the prosthesis had a reduced CoP path length and presented a smaller sway area during quiet standing. Also, they had decreased gait variability and overall improved balance confidence [33]. The effects of wearing a bone-anchored prosthesis on walking have previously been contrasted with individuals that use socket-attached prostheses, able-bodied controls as reference, and within the individuals using socket- and OI-prostheses [34–37]. It was observed that users of bone-anchored prostheses had an improved gait, as seen by a shorter gait cycle, a shorter support phase, a shorter switching phase [34], reduced hip extension, poster/anterior tilt [36], and improved frontal plane gait kinematics (observed in trunk, hip, and pelvis angle movements [35]). This might be because users of bone-anchored prostheses have patterns of muscle co-activations during walking closer to the one observed in able-bodied individuals. In another study, it was observed that users of OI and socket-based prostheses share similar gait characteristics with comparable levels of confidence in the use of their prosthesis [38]. Further, users of bone-anchored prostheses exhibited higher muscle activity associated with hip control, which might be related to compensatory movements throughout the swing cycle. In general, decreased motor activity may explain the reduced energy expenditure observed in users of bone-anchored prostheses [39]. However, another study showed a decreased ability to isometrically contract muscles of the residual limb in user of bone-anchored protheses [40]. Despite increased mobility and the other named advantages of OI, an increased risk due to the two-stage surgical interventions, periprosthetic fractures, and adverse effects at the skin-prosthesis interface, including infections, remain as possible setbacks [41,42]. Thus, there is a need to further understand how using a bone-anchored prosthesis impacts the neuromuscular control of balance during walking and standing. Previous findings showed that changes in these measures are indicative of neuromuscular changes in transfemoral amputees with socket-type prostheses [14,15].

The objective of this case study was to investigate the neuromuscular control of the trunk and the intact limb of one lower limb amputee before and after undergoing OI using electromyography (EMG) and CoP measures during upright standing. Surface EMG signals were measured six months before and one and a half years after undergoing the OI surgery to investigate the activity and co-activation of the muscles controlling the hip and the ankle of the intact limb. Simultaneously, CoP data were measured to investigate changes in the postural control of body sway [14]. We hypothesize that post-OI surgery, intermuscular coupling reflected in coherency values computed from the four muscle pairs will more closely align to control values. Similarly, EMG intensity resembling the energy content of the muscle activity will approach control levels. These results may be indicative of less stringent and more energy-efficient and balanced muscle control mechanisms. We anticipate that after the OI procedure, there will be noticeable alterations in CoP values closer to the control values, especially on the amputated side. We also predict that this will be mirrored in the dynamics of the CoP control.

## Methods

### Participants

One 52-year-old male, unilateral transfemoral amputee (height: 170 cm, weight: 83 kg, stump-length: 35 cm after surgery, distal amputation) participated in this study. He underwent a lower limb amputation of his left leg at the age of 46 due to a work-related accident. After amputation, he experienced for six years discomfort, poor fit and enormous sweating due to his socket-type prosthesis. He described that ‘the stump changed its volume over the day, consequently causing the socket to become loose’. He was using a Rheo knee prosthesis (Össur Deutschland GmbH, Köln, Germany) and later a Genium prosthesis (Otto Bock HealthCare Deutschland GmbH, Duderstadt, Germany). Due to his problems, an osseointegration surgery was advised. He was examined six months prior and one and a half years after OI and direct skeletal attachment of the prosthesis.

This study also included a control group of nine able-bodied adults (age: 55.2 ± 6.6 yrs.; height: 169 ± 9 cm; weight: 73.4 ± 11.0 kg; 6 females and 3 males). All participants had normal or corrected-to-normal vision and showed no neurological or cardiovascular diseases or other orthopedic conditions. All participants of the control group as well as the patient were informed and signed a consent form. This study was approved by the ethical commission of the University of Freiburg (ethical approval N°230/18).

### Experimental protocol and measurements

Participants were instructed to quietly stand upright with both feet at a shoulder-width distance and their arms hanging loosely at the side on a pressure platform (FDM-S, zebris Medical GmbH, Isny, Germany). They were instructed to fixate a cross at a 1.5 m distance in front of them at eye level. Each participant performed three trials for both visual conditions: eyes open and eyes closed. Each trial lasted 30 s, and the order of the trials was randomized, and all trials were separated by short breaks.

CoP position under each foot and the whole body were recorded at 60 Hz. Bipolar surface EMG signals from muscles controlling the hip and the ankle joint of the intact limb or dominant side of controls were simultaneously measured at 2 kHz (gain: 1000, hardware-filtered at 5–500 Hz) with an MP35 system (BIOPAC, CA, USA). These muscles included the M. obliquus externus, the M. erector spinae, the M. tibialis anterior, and the M. gastrocnemius medialis. The former two muscles were selected as they are involved in the hip strategy to control balance during upright standing, rotating the body around the hip [43]. The latter two muscles represent muscles that are used in the ankle strategy where the body moves around the ankle in all directions [44]. Electrodes were placed according to the SENIAM project guidelines [45]. Reference electrodes were placed on the bony part of the medial and lateral malleolus.

### EMG data analysis

To eliminate any transients between trials, the first and last 2.5 s of each measurement were discarded. A band-pass wavelet filter was applied to each EMG time series with cut-off frequencies of 2.5 and 500 Hz. The signals were notch-filtered at 50 Hz and its harmonics to remove line frequency contaminations. The EMG signals were then separated in frequency and time by applying a wavelet filter bank resulting in time series at 7 center frequencies (*cf* = 7.56, 19.86, 38.26, 62.63, 92.90, 129.00, and 170.90 Hz). The wavelet-transformed signals were then used to compute for all center frequencies the modulus of the coherency between pairs of EMG signals. The coherency provides thus a measure between 0 and 1 reflecting a proportional strength of the muscle co-activation measured by the EMG signals [15,46,47]. The significance threshold of the coherency was estimated as follows. First, a baseline threshold was computed by calculating the coherency while shifting one of the EMG signals by one sample in time and repeating this process for 200 samples, hence destroying any temporal coherence between the two EMG signals. The 200 baseline coherency values for each trial were then averaged across all trials for each participant. Finally, the actual coherency is evaluated against the baseline coherency plus two times its standard deviation, and if the coherency is above the baseline threshold it is considered strong and not a result of chance or noise in the data. EMG intensity of each muscle was also computed by applying the wavelet filter bank to each EMG signal, averaged across time, describing thus the energy density for each frequency [46].

### Data Analysis CoP measures

A bandpass wavelet filter was applied to the CoP data with cut-off frequencies at 0.15 and 10 Hz. The area, the total length, and velocity of the CoP adjustments were computed according to Prieto et al. [17] in the anterior-posterior (AP) and medio-lateral (ML) directions for both legs, the amputated and the intact leg, and the non-dominant and the dominant leg of controls. Non-linear variability of the filtered CoP signals was computed using the Entropic Half-Life method (EnHL) as previously described [9,14,15,48,49]. High EnHL values represent lower dynamics in the adjustments of the CoP, while low EnHL values represent higher dynamic CoP adjustments.

### Statistical analysis

Due to the low sample size taken from the amputee patient (three samples), only statistical analyses between the measures of the amputee patient and controls are possible. The results were analysed by using one-sample *t*-tests with Benjamini-Hochberg corrections to adjust for multiple-comparison. Statistical analysis was conducted in R (R Core Team, [50]). The mean was computed from the amputee values and tested against the control values.

## Results

### Wavelet coherency

**Figure 1.**
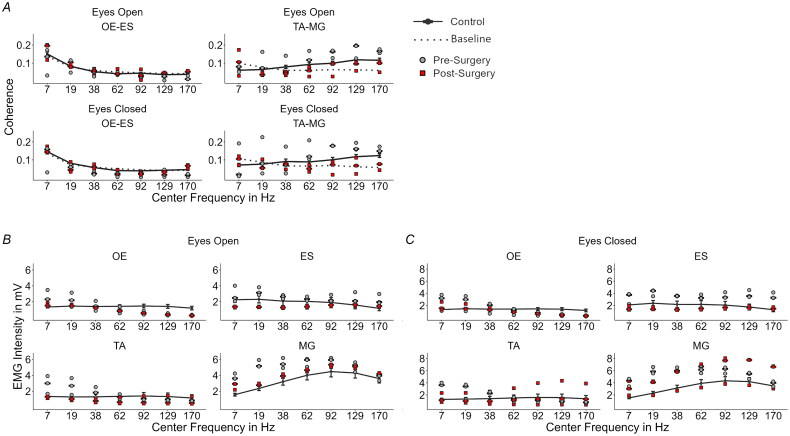
(A) EMG wavelet coherence for the two muscle Combinations OE-ES and TA-MG shown for each center frequency. Values for pre- and post-bone anchoring trials of the amputee are shown with grey-filled circles and red-filled squares respectively. The dotted line represents the baseline computed from the amputee data. The control group is shown as black dots and connected by a solid line with added error bars (standard error of the mean). (B) EMG intensity for the eyes open and (C) for the eyes closed condition for each measured muscle. (OE: obliquus externus, ES: erector spinae, TA: tibialis anterior, and MG: medialis gastrocnemius, EO: eyes open, EC: eyes closed).

Only the *obliquus externus* x *erector spinae* and *tibialis anterior* x *gastrocnemius medialis* muscle pairs showed significant strong coupling with respect to the baseline threshold (Figure 1A). Further, *obliquus externus x erector spinae* only showed differences for the post-bone-anchoring of the prosthesis condition with eyes open at 7 Hz (*t* = 3.32; *p* = .004) and 170 Hz (*t* = 5.07, *p* < .001) with respect to baseline and the control group (Figure 1A), where coherency values pre-bone anchoring of the prosthesis decreased well below the baseline. With eyes closed, this was only observed at 170 Hz (post-OI: *t* = 6.31, *p* < .001, Figure 1A). For the muscle pair *tibialis anterior* x *gastrocnemius medialis*, the differences were more pronounced over a wider frequency range (Figure 1A). For both visual conditions before bone anchoring, the coherency values within the frequency range of 62–170 Hz were higher than the values of controls (Eyes Open: 62 Hz: *t* = 3.31, *p* = .003, 92 Hz: *t* = 2.30, *p* = .029, 129 Hz: *t* = 2.67, *p* = .0129, 170 Hz: *t* = 2.53, *p* = .018; Eyes Closed: 62 Hz: *t* = 2.50, *p* = .036, 92 Hz: *t* = 3.14, *p* = .011, 129 Hz: *t* = 4.17, *p* = .003, 170 Hz: *t* = 3.39, *p* = .008). After the bone anchoring of the prosthesis, the coherency values were reduced to the level of the control group and for some trials to below the baseline threshold (Figure 1A, see Supplementary Table S1).

### EMG intensity

With the eyes opened and before skeletal attachment of the prosthesis, the amputee patient showed increased EMG intensity values compared to controls in the lower to high-frequency range for the muscles *obliquus externus* (7 Hz: *t* = −9.52, *p* < .001, 19 Hz: *t* = −6.70, *p* < .001), *tibialis anterior* (7 Hz: *t* = −7.35, *p* < .001, 19 Hz: *t* = −5.91, *p* < .001), and *gastrocnemius medialis* (7 Hz: *t* = −12.77, *p* < .001, 19 Hz: *t* = −11.33, *p* < .001, 38 Hz: *t* = −5.27, *p* < .001, 62 Hz: *t* = −2.89, *p* = .021) (Figure 1B).

After the skeletal attachment of the prosthesis, these differences were reduced (Figure 1B, see Supplementary Table S2). While the same pattern was observed for the muscle *erector spinae*, the differences with respect to the control group were smaller, especially before OI (pre-OI at 170 Hz: *t* = −3.61, *p* = .004). For this muscle, the EMG intensity was even smaller than in controls after bone anchoring of the prosthesis (post-OI: 17 Hz. *t* = 2.71, *p* = .03). During the eyes closed condition, the same behaviour for the muscles *obliquus externus, tibialis anterior*, and *erector spinae* was observed. The differences for the *erector spinae* muscle pair were more pronounced during the eyes closed condition (Figure 1C). The pre-OI values were all significantly higher than the control values (pre-OI: 7 Hz: *t* = −4.24, *p* = .002, 38 Hz: *t* = −2.57, *p* = .039, 92 Hz: *t* = −2.54, *p* = .04, 129 Hz: *t* = −4.32, *p* = .001, 170 Hz: *t* = −6.99, *p* < .001), and the post-OI values did not show any significant difference from the control group (see Supplementary Table S2). Interestingly, *gastrocnemius medialis* presented increased EMG intensity after bone anchoring of the prosthesis at the whole frequency range (post-OI: 7 Hz: *t* = −11.65, *p* < .001, 19 Hz: *t* = −3.99, *p* = .002, 38 Hz: *t* = −3.63, *p* = .004, 62 Hz: *t* = −2.86, *p* = .022, 92 Hz: *t* = −3.23, *p* = .009, 129 Hz: *t* = −3.36, *p* = .009, 170 Hz: *t* = −3.69, *p* = .003).

### CoP results

**Figure 2.**
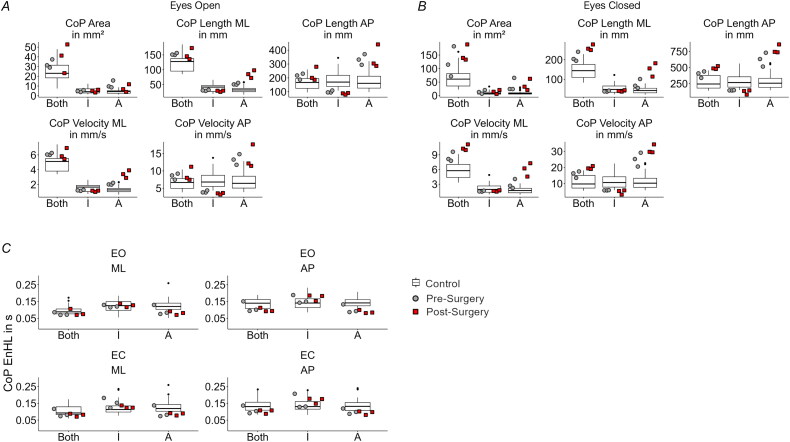
(A) Sway measures for the eyes open and (B) for the eyes closed condition. (C) EnHL in seconds for the different directions and conditions. The grey-filled circles and the red-filled squares are the respective values for each trial for the amputee participant. The control results are shown in box plots. I: Intact leg, for the controls dominant leg; A: Amputated leg, for the control non-dominant leg; ML: medio-lateral; AP: anterior-posterior; EO: eyes open; EC: eyes closed.

The statistical analysis revealed that most of the sway and EnHL values pre- and post-surgery were different between the amputee patient and controls (see Supplementary Tables S3 and S4). The sway area increased when the visual information was removed, and the amputee presented for both pre- and post-bone anchoring of the prosthesis an increased sway area compared to controls (Figure 2A,B, see Supplementary Table S3). This was not the case only for pre- and post-surgery during the eyes open condition for the intact leg (pre: *t* = −0.04, *p* = .969, post: *t* = −0.25, *p* = .811). There were no larger differences observed pre- and post-bone anchoring of the prosthesis for both the intact and the amputated leg. Thus, OI for this patient did not have a strong effect on the overall area of the CoP. The total length of the CoP of the amputated side increased for the amputee patient post-bone anchoring of the prosthesis in the ML direction. Closing the eyes marginally increased the spread in both legs pre-bone anchoring of the prosthesis in the ML direction. The CoP velocity was mainly increased for the eyes closed condition for the amputated leg and both legs after the bone-anchoring (Figure 2A,B).

At last, the EnHL values showed smaller differences in the CoP dynamics before and after the bone anchoring of the prosthesis (Figure 2C). The EnHL in the amputee patient did not show vast changes from controls (see Supplementary Table S4). Most of the EnHL values pre- and post-OI were different from controls, except for the ones from the intact leg during the eyes open condition in the ML direction (pre: *t* = 0.25, *p* = .803, post: *t* = −0.57, *p* = .599), as well as the ones from the intact leg during the eyes closed condition in the ML direction post-surgery (*t* = −0.61, *p* = .598), and in the AP direction pre-surgery (*t* = −1.58, *p* = .145).

## Discussion

This study sought to investigate the impact of osseointegration allowing for direct skeletal attachment of the prosthesis on the neuromuscular system during upright standing in one participant with a unilateral transfemoral amputation. EMG signals were used to compute the coupling strength and activation intensity of lower body muscles, and CoP data were used to investigate sway parameters and the dynamics of sway adjustments.

The participant presented significant coupling only in the muscle pairs of *obliquus externus* x *erector spinae* and *tibialis anterior* x *gastrocnemius medialis*, which was also observed in a previous study with amputees wearing socket-type prostheses [15]. The differences in EMG activation observed at the low frequencies have been previously associated with unconscious balance control [51], suggesting that a larger part of the neuromuscular control over balance may be controlled sub-cortically after the osseointegration. The muscle pair controlling the ankle showed for both visual conditions decreased coherency values after OI and direct bone anchoring of the prosthesis at high frequencies (62–170 Hz). At this frequency range, EMG activity is associated with fast oscillations of motor units from nerves with high conduction velocities [52] and changes in the shape of compound muscle action potentials [53,54]. The higher co-activation strength may indicate that before OI, the participant relied on a more rigid control over the intact leg muscles to maintain balance. The central nervous system selects task-specific fast and slow conducting muscle fibers [55], where fast twitching fibers are believed to generate more frequent motor unit action potentials and are associated with the ability to produce faster force adjustments to perturbations [56]. This may be observed more in the *tibialis anterior* x *gastrocnemius medialis* pair rather than in the trunk muscles because they are used with a higher proportion during normal standing. Thus, the change to normal levels after OI and following bone-anchoring of the prosthesis suggests a reduced need for the *tibialis anterior* x *gastrocnemius medialis* muscle pair.

The EMG intensity spectrum of the participant before bone anchoring of the prosthesis presented increased values at the lower frequencies for the muscles *obliquus externus* and *tibialis anterior*. This may again indicate the influence of unconscious balance control over the EMG activity. *Erector spinae* showed overall higher EMG intensity before the direct attachment of the prosthesis to the osseointegrated implant. After bone-anchoring the prosthesis, the values were even lower than the ones of the control group. Finally, the *gastrocnemius medialis* showed similar behaviour as the *obliquus externus* and *tibialis anterior* muscles except for the eyes closed condition where the intensity even rose above frequencies higher than 62 Hz after bone-anchoring of the prosthesis. Together with the coherence results, this possibly indicates a changed neuromuscular control during standing, which might be caused by increased stability, reduced compensatory movements of the RL (hip circumduction), and increased sensory feedback. This is consistent with similar studies on muscle activity in people wearing OI leg prostheses. Although previous studies investigated the activity of the residual limb, they found that walking involved highly cyclical muscle activity, with more pronounced activity in the hip muscles [40,57]. While this refers to EMG activity during walking, it still goes in line with the findings of this case report, meaning that osseointegration restores functionality as reflected in muscle activity more similar to the one of able-bodied individuals.

The CoP data showed more pronounced differences in the sway of the amputated side, with a specifically increased length and velocity of the CoP adjustments in the ML direction. These values were smaller when using the socket-type prosthesis, indicating higher mechanical stiffness in the prosthetic leg. With the OI prosthesis, the CoP length and velocity on the amputated side increased, which suggests again less stiffness bone-anchoring of the prosthesis. This is contrary to the findings of Gaffney et al. who observed reduced values in CoP path length for patients who used an osseointegrated prosthesis [33]. Measurements of pre- and post-bone-anchoring dynamics of the CoP indicate that the time-scale of the control of the CoP adjustments did not change markedly before and after the OI surgeries. CoP EnHL results are in line with findings by our earlier study where we investigated transfemoral amputees that used a socket prosthesis, except for the amputated leg, where we found faster CoP dynamics across all directions, visual and surgery conditions [14].

It must be considered that there are confounders in EMG and in the CoP recordings [58]. For EMG, it was shown that there are several factors that influence the effectiveness of the recording. One such factor is the changing position of muscles and tissues under the skin where electrodes are placed, leading to changing recordings of muscle activity. Another issue is that some muscles used for stabilization or movement can interfere with the EMG signals. A further factor can be contraction intensity, which causes variation in muscle activation patterns for the same motion under different loads. Additionally, changes in electrode position affect the measured signal due to the shift of the underlying musculature and changes in contact impedance between the skin and the electrodes. Finally, there is other noise sources that can influence the signal stemming from electrical devices or other movements and from the different positioning of the electrodes by the experimenter [58]. For CoP data, confounders include age, gender, body mass index, and physical activity level. All these variables affect the accuracy of CoP measurements and should be considered when interpreting the results [59]. While we controlled for age in the current study, this study did not account for the other factors as this was a case study. In future studies, it should be considered to aim for gender-balanced or more targeted participant selection.

## Conclusions

The results of this study indicate changes in the neuromuscular control over balance in an amputee after undergoing OI. Intermuscular coupling and EMG intensity of four muscles involved in balance control showed trends approaching able-bodied baseline levels. CoP data also indicate changes in balance control, pointing to more active use of the prosthetic leg after OI and further away from control values. Taken together, a preliminary picture was captured of what happens after OI in a patient with a transfemoral unilateral amputation, and how the neuromuscular control system is affected by this procedure. These results need further confirmation with a larger population of participants with an amputation.

## Supplementary Material

Supplemental Material

## Data Availability

The data used in this work is available upon reasonable request to the corresponding author.
