# Supplementary material for: Neuromuscular adaptations after osseointegration of a bone-anchored prosthesis in a unilateral transfemoral amputee – a case study
Source: Ann Med. 2023 Sep 7;55(2):2255206. doi: 10.1080/07853890.2023.2255206 (PMC10486294; doi:10.1080/07853890.2023.2255206)
Supplement: Supplemental Material [file IANN_A_2255206_SM7053.docx]

Neuromuscular Adaptations after Osseointegration of a Bone-Anchored Prosthesis in a Unilateral Transfemoral Amputee – a Case Study

T. Krauskopf^1,2^, T. B. Lauck^1^, B. Meyer^1^, L. Klein^3^, M. Mueller^4^, E. J. Kubosch^3^, G.W. Herget^3^, V. von Tscharner^5^, J. Ernst^6,7^, T. Stieglitz^1,2,8^ and C. Pasluosta^1,2*^

**Supplementary Material**

Table S1. Statistical analysis of the Coherency results of the one-sample-t-test with Benjamini-Hochberg correction. Significance at α = 0.05, Muscle abbreviations are: OE: obliquus externus, ES: erector spinae, TA: tibials anterior, and MG: medial gastrocnemius.

| **Condition** | **MusclePair** | **CenterFrequency** | **Surgery** | **T-Value** | **p-Value** | **adjusted-p-Value** |
| --- | --- | --- | --- | --- | --- | --- |
| Eyes Open | OE-ES | 7 | Pre-Surgery | 3.324 | 0.003 | 0.004 |
| Eyes Open | OE-ES | 19 | Pre-Surgery | -1.312 | 0.201 | 0.245 |
| Eyes Open | OE-ES | 38 | Pre-Surgery | 0.988 | 0.332 | 0.388 |
| Eyes Open | OE-ES | 62 | Pre-Surgery | 0.040 | 0.969 | 0.969 |
| Eyes Open | OE-ES | 92 | Pre-Surgery | 4.479 | 0.000 | 0.000 |
| Eyes Open | OE-ES | 129 | Pre-Surgery | 3.861 | 0.001 | 0.001 |
| Eyes Open | OE-ES | 170 | Pre-Surgery | 5.070 | 0.000 | 0.000 |
| Eyes Open | TA-MG | 7 | Pre-Surgery | -1.537 | 0.137 | 0.174 |
| Eyes Open | TA-MG | 19 | Pre-Surgery | -3.336 | 0.003 | 0.004 |
| Eyes Open | TA-MG | 38 | Pre-Surgery | -0.198 | 0.844 | 0.937 |
| Eyes Open | TA-MG | 62 | Pre-Surgery | -2.338 | 0.027 | 0.037 |
| Eyes Open | TA-MG | 92 | Pre-Surgery | -4.822 | 0.000 | 0.000 |
| Eyes Open | TA-MG | 129 | Pre-Surgery | -4.519 | 0.000 | 0.000 |
| Eyes Open | TA-MG | 170 | Pre-Surgery | -4.271 | 0.000 | 0.001 |
| Eyes Closed | OE-ES | 7 | Pre-Surgery | 2.483 | 0.020 | 0.028 |
| Eyes Closed | OE-ES | 19 | Pre-Surgery | 3.791 | 0.001 | 0.002 |
| Eyes Closed | OE-ES | 38 | Pre-Surgery | 4.926 | 0.000 | 0.000 |
| Eyes Closed | OE-ES | 62 | Pre-Surgery | 4.740 | 0.000 | 0.000 |
| Eyes Closed | OE-ES | 92 | Pre-Surgery | 4.339 | 0.000 | 0.001 |
| Eyes Closed | OE-ES | 129 | Pre-Surgery | 4.687 | 0.000 | 0.000 |
| Eyes Closed | OE-ES | 170 | Pre-Surgery | 6.314 | 0.000 | 0.000 |
| Eyes Closed | TA-MG | 7 | Pre-Surgery | -0.166 | 0.870 | 0.937 |
| Eyes Closed | TA-MG | 19 | Pre-Surgery | -2.788 | 0.010 | 0.014 |
| Eyes Closed | TA-MG | 38 | Pre-Surgery | 0.109 | 0.914 | 0.948 |
| Eyes Closed | TA-MG | 62 | Pre-Surgery | -3.788 | 0.001 | 0.002 |
| Eyes Closed | TA-MG | 92 | Pre-Surgery | -5.949 | 0.000 | 0.000 |
| Eyes Closed | TA-MG | 129 | Pre-Surgery | -4.427 | 0.000 | 0.000 |
| Eyes Closed | TA-MG | 170 | Pre-Surgery | -3.123 | 0.004 | 0.007 |
| Eyes Open | OE-ES | 7 | Post-Surgery | -1.882 | 0.071 | 0.095 |
| Eyes Open | OE-ES | 19 | Post-Surgery | -0.391 | 0.699 | 0.725 |
| Eyes Open | OE-ES | 38 | Post-Surgery | -0.271 | 0.789 | 0.789 |
| Eyes Open | OE-ES | 62 | Post-Surgery | -1.511 | 0.143 | 0.174 |
| Eyes Open | OE-ES | 92 | Post-Surgery | 2.030 | 0.053 | 0.074 |
| Eyes Open | OE-ES | 129 | Post-Surgery | -2.026 | 0.053 | 0.074 |
| Eyes Open | OE-ES | 170 | Post-Surgery | -3.780 | 0.001 | 0.004 |
| Eyes Open | TA-MG | 7 | Post-Surgery | -6.091 | 0.000 | 0.000 |
| Eyes Open | TA-MG | 19 | Post-Surgery | 3.269 | 0.003 | 0.009 |
| Eyes Open | TA-MG | 38 | Post-Surgery | 4.041 | 0.000 | 0.003 |
| Eyes Open | TA-MG | 62 | Post-Surgery | 3.314 | 0.003 | 0.008 |
| Eyes Open | TA-MG | 92 | Post-Surgery | 2.304 | 0.029 | 0.049 |
| Eyes Open | TA-MG | 129 | Post-Surgery | 2.671 | 0.013 | 0.028 |
| Eyes Open | TA-MG | 170 | Post-Surgery | 2.535 | 0.018 | 0.035 |
| Eyes Closed | OE-ES | 7 | Post-Surgery | -0.636 | 0.530 | 0.594 |
| Eyes Closed | OE-ES | 19 | Post-Surgery | 3.961 | 0.001 | 0.003 |
| Eyes Closed | OE-ES | 38 | Post-Surgery | -0.895 | 0.379 | 0.442 |
| Eyes Closed | OE-ES | 62 | Post-Surgery | 2.390 | 0.024 | 0.043 |
| Eyes Closed | OE-ES | 92 | Post-Surgery | 3.975 | 0.001 | 0.003 |
| Eyes Closed | OE-ES | 129 | Post-Surgery | 2.271 | 0.032 | 0.049 |
| Eyes Closed | OE-ES | 170 | Post-Surgery | -3.462 | 0.002 | 0.008 |
| Eyes Closed | TA-MG | 7 | Post-Surgery | -3.088 | 0.005 | 0.011 |
| Eyes Closed | TA-MG | 19 | Post-Surgery | 0.461 | 0.649 | 0.698 |
| Eyes Closed | TA-MG | 38 | Post-Surgery | 1.646 | 0.112 | 0.142 |
| Eyes Closed | TA-MG | 62 | Post-Surgery | 2.498 | 0.019 | 0.036 |
| Eyes Closed | TA-MG | 92 | Post-Surgery | 3.141 | 0.004 | 0.011 |
| Eyes Closed | TA-MG | 129 | Post-Surgery | 4.175 | 0.000 | 0.003 |
| Eyes Closed | TA-MG | 170 | Post-Surgery | 3.390 | 0.002 | 0.008 |

Table S2. Statistical analysis of the EMG-intensity results of the one-sample-t-test with Benjamini-Hochberg correction. Significance at α = 0.05, Muscle abbreviations are: OE: obliquus externus, ES: erector spinae, TA: tibials anterior, and MG: medial gastrocnemius.

| **Condition** | **Frequency** | **Muscle** | **Surgery** | **T-Value** | **p-Value** | **Adjusted-p-Value** |
| --- | --- | --- | --- | --- | --- | --- |
| Eyes Open | 7 | ES | Pre-Surgery | -1,814 | 0,081 | 0,149 |
| Eyes Open | 19 | ES | Pre-Surgery | -2,141 | 0,042 | 0,092 |
| Eyes Open | 38 | ES | Pre-Surgery | -0,956 | 0,348 | 0,453 |
| Eyes Open | 62 | ES | Pre-Surgery | -0,514 | 0,612 | 0,678 |
| Eyes Open | 92 | ES | Pre-Surgery | -0,763 | 0,452 | 0,544 |
| Eyes Open | 129 | ES | Pre-Surgery | -1,818 | 0,081 | 0,149 |
| Eyes Open | 170 | ES | Pre-Surgery | -3,608 | 0,001 | 0,004 |
| Eyes Closed | 7 | ES | Pre-Surgery | -4,240 | 0,000 | 0,001 |
| Eyes Closed | 19 | ES | Pre-Surgery | -3,871 | 0,001 | 0,002 |
| Eyes Closed | 38 | ES | Pre-Surgery | -2,570 | 0,016 | 0,039 |
| Eyes Closed | 62 | ES | Pre-Surgery | -1,994 | 0,057 | 0,113 |
| Eyes Closed | 92 | ES | Pre-Surgery | -2,545 | 0,017 | 0,040 |
| Eyes Closed | 129 | ES | Pre-Surgery | -4,320 | 0,000 | 0,001 |
| Eyes Closed | 170 | ES | Pre-Surgery | -6,997 | 0,000 | 0,000 |
| Eyes Open | 7 | MG | Pre-Surgery | -12,774 | 0,000 | 0,000 |
| Eyes Open | 19 | MG | Pre-Surgery | -11,329 | 0,000 | 0,000 |
| Eyes Open | 38 | MG | Pre-Surgery | -5,274 | 0,000 | 0,000 |
| Eyes Open | 62 | MG | Pre-Surgery | -2,890 | 0,008 | 0,021 |
| Eyes Open | 92 | MG | Pre-Surgery | -1,911 | 0,067 | 0,129 |
| Eyes Open | 129 | MG | Pre-Surgery | -1,254 | 0,221 | 0,339 |
| Eyes Open | 170 | MG | Pre-Surgery | -0,462 | 0,648 | 0,712 |
| Eyes Closed | 7 | MG | Pre-Surgery | -19,476 | 0,000 | 0,000 |
| Eyes Closed | 19 | MG | Pre-Surgery | -13,126 | 0,000 | 0,000 |
| Eyes Closed | 38 | MG | Pre-Surgery | -6,375 | 0,000 | 0,000 |
| Eyes Closed | 62 | MG | Pre-Surgery | -3,710 | 0,001 | 0,003 |
| Eyes Closed | 92 | MG | Pre-Surgery | -2,628 | 0,014 | 0,035 |
| Eyes Closed | 129 | MG | Pre-Surgery | -1,612 | 0,119 | 0,202 |
| Eyes Closed | 170 | MG | Pre-Surgery | -0,691 | 0,496 | 0,578 |
| Eyes Open | 7 | OE | Pre-Surgery | -9,525 | 0,000 | 0,000 |
| Eyes Open | 19 | OE | Pre-Surgery | -6,702 | 0,000 | 0,000 |
| Eyes Open | 38 | OE | Pre-Surgery | -0,448 | 0,658 | 0,716 |
| Eyes Open | 62 | OE | Pre-Surgery | 2,613 | 0,015 | 0,036 |
| Eyes Open | 92 | OE | Pre-Surgery | 3,821 | 0,001 | 0,003 |
| Eyes Open | 129 | OE | Pre-Surgery | 4,128 | 0,000 | 0,002 |
| Eyes Open | 170 | OE | Pre-Surgery | 4,100 | 0,000 | 0,002 |
| Eyes Closed | 7 | OE | Pre-Surgery | -12,889 | 0,000 | 0,000 |
| Eyes Closed | 19 | OE | Pre-Surgery | -9,141 | 0,000 | 0,000 |
| Eyes Closed | 38 | OE | Pre-Surgery | -1,739 | 0,094 | 0,165 |
| Eyes Closed | 62 | OE | Pre-Surgery | 2,001 | 0,056 | 0,113 |
| Eyes Closed | 92 | OE | Pre-Surgery | 3,477 | 0,002 | 0,005 |
| Eyes Closed | 129 | OE | Pre-Surgery | 3,888 | 0,001 | 0,002 |
| Eyes Closed | 170 | OE | Pre-Surgery | 3,900 | 0,001 | 0,002 |
| Eyes Open | 7 | TA | Pre-Surgery | -7,345 | 0,000 | 0,000 |
| Eyes Open | 19 | TA | Pre-Surgery | -5,907 | 0,000 | 0,000 |
| Eyes Open | 38 | TA | Pre-Surgery | -1,847 | 0,076 | 0,145 |
| Eyes Open | 62 | TA | Pre-Surgery | 0,124 | 0,902 | 0,931 |
| Eyes Open | 92 | TA | Pre-Surgery | 0,751 | 0,460 | 0,547 |
| Eyes Open | 129 | TA | Pre-Surgery | 0,889 | 0,382 | 0,481 |
| Eyes Open | 170 | TA | Pre-Surgery | 0,791 | 0,436 | 0,531 |
| Eyes Closed | 7 | TA | Pre-Surgery | -9,166 | 0,000 | 0,000 |
| Eyes Closed | 19 | TA | Pre-Surgery | -5,507 | 0,000 | 0,000 |
| Eyes Closed | 38 | TA | Pre-Surgery | -1,484 | 0,150 | 0,243 |
| Eyes Closed | 62 | TA | Pre-Surgery | 0,406 | 0,688 | 0,734 |
| Eyes Closed | 92 | TA | Pre-Surgery | 1,002 | 0,326 | 0,439 |
| Eyes Closed | 129 | TA | Pre-Surgery | 1,145 | 0,263 | 0,373 |
| Eyes Closed | 170 | TA | Pre-Surgery | 1,144 | 0,263 | 0,373 |
| Eyes Open | 7 | ES | Post-Surgery | 2,710 | 0,012 | 0,030 |
| Eyes Open | 19 | ES | Post-Surgery | 2,117 | 0,044 | 0,093 |
| Eyes Open | 38 | ES | Post-Surgery | 1,448 | 0,160 | 0,255 |
| Eyes Open | 62 | ES | Post-Surgery | 1,054 | 0,301 | 0,412 |
| Eyes Open | 92 | ES | Post-Surgery | 0,543 | 0,592 | 0,663 |
| Eyes Open | 129 | ES | Post-Surgery | -0,061 | 0,952 | 0,952 |
| Eyes Open | 170 | ES | Post-Surgery | -0,910 | 0,371 | 0,478 |
| Eyes Closed | 7 | ES | Post-Surgery | 2,002 | 0,056 | 0,113 |
| Eyes Closed | 19 | ES | Post-Surgery | 1,919 | 0,066 | 0,129 |
| Eyes Closed | 38 | ES | Post-Surgery | 1,554 | 0,132 | 0,218 |
| Eyes Closed | 62 | ES | Post-Surgery | 1,175 | 0,251 | 0,373 |
| Eyes Closed | 92 | ES | Post-Surgery | 0,728 | 0,473 | 0,558 |
| Eyes Closed | 129 | ES | Post-Surgery | 0,155 | 0,878 | 0,919 |
| Eyes Closed | 170 | ES | Post-Surgery | -0,645 | 0,524 | 0,605 |
| Eyes Open | 7 | MG | Post-Surgery | -6,308 | 0,000 | 0,000 |
| Eyes Open | 19 | MG | Post-Surgery | -1,781 | 0,087 | 0,157 |
| Eyes Open | 38 | MG | Post-Surgery | -1,737 | 0,094 | 0,165 |
| Eyes Open | 62 | MG | Post-Surgery | -1,058 | 0,300 | 0,412 |
| Eyes Open | 92 | MG | Post-Surgery | -1,172 | 0,252 | 0,373 |
| Eyes Open | 129 | MG | Post-Surgery | -1,169 | 0,253 | 0,373 |
| Eyes Open | 170 | MG | Post-Surgery | -1,061 | 0,298 | 0,412 |
| Eyes Closed | 7 | MG | Post-Surgery | -11,651 | 0,000 | 0,000 |
| Eyes Closed | 19 | MG | Post-Surgery | -3,997 | 0,000 | 0,002 |
| Eyes Closed | 38 | MG | Post-Surgery | -3,627 | 0,001 | 0,004 |
| Eyes Closed | 62 | MG | Post-Surgery | -2,856 | 0,008 | 0,022 |
| Eyes Closed | 92 | MG | Post-Surgery | -3,231 | 0,003 | 0,009 |
| Eyes Closed | 129 | MG | Post-Surgery | -3,361 | 0,002 | 0,007 |
| Eyes Closed | 170 | MG | Post-Surgery | -3,697 | 0,001 | 0,003 |
| Eyes Open | 7 | OE | Post-Surgery | -2,121 | 0,044 | 0,093 |
| Eyes Open | 19 | OE | Post-Surgery | -0,561 | 0,580 | 0,656 |
| Eyes Open | 38 | OE | Post-Surgery | 0,983 | 0,335 | 0,446 |
| Eyes Open | 62 | OE | Post-Surgery | 2,806 | 0,009 | 0,024 |
| Eyes Open | 92 | OE | Post-Surgery | 3,836 | 0,001 | 0,003 |
| Eyes Open | 129 | OE | Post-Surgery | 4,201 | 0,000 | 0,001 |
| Eyes Open | 170 | OE | Post-Surgery | 4,118 | 0,000 | 0,002 |
| Eyes Closed | 7 | OE | Post-Surgery | -4,576 | 0,000 | 0,001 |
| Eyes Closed | 19 | OE | Post-Surgery | -2,244 | 0,034 | 0,075 |
| Eyes Closed | 38 | OE | Post-Surgery | 0,169 | 0,867 | 0,916 |
| Eyes Closed | 62 | OE | Post-Surgery | 2,460 | 0,021 | 0,048 |
| Eyes Closed | 92 | OE | Post-Surgery | 3,566 | 0,001 | 0,004 |
| Eyes Closed | 129 | OE | Post-Surgery | 4,041 | 0,000 | 0,002 |
| Eyes Closed | 170 | OE | Post-Surgery | 4,004 | 0,000 | 0,002 |
| Eyes Open | 7 | TA | Post-Surgery | 0,430 | 0,671 | 0,722 |
| Eyes Open | 19 | TA | Post-Surgery | 1,332 | 0,195 | 0,303 |
| Eyes Open | 38 | TA | Post-Surgery | 1,689 | 0,103 | 0,178 |
| Eyes Open | 62 | TA | Post-Surgery | 1,571 | 0,128 | 0,215 |
| Eyes Open | 92 | TA | Post-Surgery | 1,349 | 0,189 | 0,298 |
| Eyes Open | 129 | TA | Post-Surgery | 1,147 | 0,262 | 0,373 |
| Eyes Open | 170 | TA | Post-Surgery | 0,974 | 0,339 | 0,447 |
| Eyes Closed | 7 | TA | Post-Surgery | -0,861 | 0,397 | 0,490 |
| Eyes Closed | 19 | TA | Post-Surgery | -0,090 | 0,929 | 0,937 |
| Eyes Closed | 38 | TA | Post-Surgery | 0,108 | 0,915 | 0,931 |
| Eyes Closed | 62 | TA | Post-Surgery | -0,112 | 0,912 | 0,931 |
| Eyes Closed | 92 | TA | Post-Surgery | -0,566 | 0,576 | 0,656 |
| Eyes Closed | 129 | TA | Post-Surgery | -0,859 | 0,398 | 0,490 |
| Eyes Closed | 170 | TA | Post-Surgery | -0,896 | 0,379 | 0,481 |

Table S3: Statistical analysis of the Sway Parameter results of the one-sample-t-test with Benjamini-Hochberg correction. Significance at α = 0.05. Abbreviations: ML: Medio-lateral, AP: Anterior-posterior

| **Condition** | **Parameter** | **Side** | **Surgery** | **T-value** | **p-Value** | **Adjusted-p-Value** |
| --- | --- | --- | --- | --- | --- | --- |
| Eyes Open | Sway Area | Both | Pre-Surgery | -3.460 | 0.002 | 0.002 |
| Eyes Open | Sway Area | Amputated | Pre-Surgery | -17.139 | 0.000 | 0.000 |
| Eyes Open | Sway Area | Intact | Pre-Surgery | -0.039 | 0.969 | 0.969 |
| Eyes Open | Sway Length ML | Both | Pre-Surgery | -5.194 | 0.000 | 0.000 |
| Eyes Open | Sway Length ML | Amputated | Pre-Surgery | -8.645 | 0.000 | 0.000 |
| Eyes Open | Sway Length ML | Intact | Pre-Surgery | 3.410 | 0.002 | 0.003 |
| Eyes Open | Sway Length AP | Both | Pre-Surgery | -5.527 | 0.000 | 0.000 |
| Eyes Open | Sway Length AP | Amputated | Pre-Surgery | -16.111 | 0.000 | 0.000 |
| Eyes Open | Sway Length AP | Intact | Pre-Surgery | 6.982 | 0.000 | 0.000 |
| Eyes Open | Sway Velocity ML | Both | Pre-Surgery | -5.194 | 0.000 | 0.000 |
| Eyes Open | Sway Velocity ML | Amputated | Pre-Surgery | -8.645 | 0.000 | 0.000 |
| Eyes Open | Sway Velocity ML | Intact | Pre-Surgery | 3.410 | 0.002 | 0.003 |
| Eyes Closed | Sway Area | Both | Pre-Surgery | -8.120 | 0.000 | 0.000 |
| Eyes Closed | Sway Area | Amputated | Pre-Surgery | -23.249 | 0.000 | 0.000 |
| Eyes Closed | Sway Area | Intact | Pre-Surgery | -2.400 | 0.024 | 0.025 |
| Eyes Closed | Sway Length ML | Both | Pre-Surgery | -8.426 | 0.000 | 0.000 |
| Eyes Closed | Sway Length ML | Amputated | Pre-Surgery | -9.739 | 0.000 | 0.000 |
| Eyes Closed | Sway Length ML | Intact | Pre-Surgery | 2.928 | 0.007 | 0.008 |
| Eyes Closed | Sway Length AP | Both | Pre-Surgery | -5.991 | 0.000 | 0.000 |
| Eyes Closed | Sway Length AP | Amputated | Pre-Surgery | -14.594 | 0.000 | 0.000 |
| Eyes Closed | Sway Length AP | Intact | Pre-Surgery | 6.622 | 0.000 | 0.000 |
| Eyes Closed | Sway Velocity ML | Both | Pre-Surgery | -8.426 | 0.000 | 0.000 |
| Eyes Closed | Sway Velocity ML | Amputated | Pre-Surgery | -9.739 | 0.000 | 0.000 |
| Eyes Closed | Sway Velocity ML | Intact | Pre-Surgery | 2.928 | 0.007 | 0.008 |
| Eyes Open | Sway Area | Both | Post-Surgery | -6.643 | 0.000 | 0.000 |
| Eyes Open | Sway Area | Amputated | Post-Surgery | -7.826 | 0.000 | 0.000 |
| Eyes Open | Sway Area | Intact | Post-Surgery | -0.250 | 0.805 | 0.822 |
| Eyes Open | Sway Length ML | Both | Post-Surgery | -5.016 | 0.000 | 0.000 |
| Eyes Open | Sway Length ML | Amputated | Post-Surgery | -24.036 | 0.000 | 0.000 |
| Eyes Open | Sway Length ML | Intact | Post-Surgery | 4.372 | 0.000 | 0.000 |
| Eyes Open | Sway Length AP | Both | Post-Surgery | -6.775 | 0.000 | 0.000 |
| Eyes Open | Sway Length AP | Amputated | Post-Surgery | -17.255 | 0.000 | 0.000 |
| Eyes Open | Sway Length AP | Intact | Post-Surgery | 8.061 | 0.000 | 0.000 |
| Eyes Open | Sway Velocity ML | Both | Post-Surgery | -5.016 | 0.000 | 0.000 |
| Eyes Open | Sway Velocity ML | Amputated | Post-Surgery | -24.036 | 0.000 | 0.000 |
| Eyes Open | Sway Velocity ML | Intact | Post-Surgery | 4.372 | 0.000 | 0.000 |
| Eyes Closed | Sway Area | Both | Post-Surgery | -12.525 | 0.000 | 0.000 |
| Eyes Closed | Sway Area | Amputated | Post-Surgery | -23.760 | 0.000 | 0.000 |
| Eyes Closed | Sway Area | Intact | Post-Surgery | -2.561 | 0.017 | 0.018 |
| Eyes Closed | Sway Length ML | Both | Post-Surgery | -14.449 | 0.000 | 0.000 |
| Eyes Closed | Sway Length ML | Amputated | Post-Surgery | -34.008 | 0.000 | 0.000 |
| Eyes Closed | Sway Length ML | Intact | Post-Surgery | 2.580 | 0.016 | 0.017 |
| Eyes Closed | Sway Length AP | Both | Post-Surgery | -10.983 | 0.000 | 0.000 |
| Eyes Closed | Sway Length AP | Amputated | Post-Surgery | -21.213 | 0.000 | 0.000 |
| Eyes Closed | Sway Length AP | Intact | Post-Surgery | 7.767 | 0.000 | 0.000 |
| Eyes Closed | Sway Velocity ML | Both | Post-Surgery | -14.449 | 0.000 | 0.000 |
| Eyes Closed | Sway Velocity ML | Amputated | Post-Surgery | -34.008 | 0.000 | 0.000 |
| Eyes Closed | Sway Velocity ML | Intact | Post-Surgery | 2.580 | 0.016 | 0.017 |

Table S4: Statistical analysis of the EnHL of the CoP results of the one-sample-t-test with Benjamini-Hochberg correction. Significance at α = 0.05. Abbreviations: ML: Medio-lateral, AP: Anterior-posterior

| **Condition** | **Direction** | **Side** | **Surgery** | **T-Value** | **p-Value** | **Adjusted-p-Value** |
| --- | --- | --- | --- | --- | --- | --- |
| Eyes Open | ML | Both | Pre-Surgery | 3.848 | 0.001 | 0.001 |
| Eyes Open | ML | Amputated | Pre-Surgery | 3.361 | 0.002 | 0.004 |
| Eyes Open | ML | Intact | Pre-Surgery | 0.252 | 0.803 | 0.803 |
| Eyes Open | AP | Both | Pre-Surgery | 3.993 | 0.000 | 0.001 |
| Eyes Open | AP | Amputated | Pre-Surgery | 6.888 | 0.000 | 0.000 |
| Eyes Open | AP | Intact | Pre-Surgery | -2.469 | 0.020 | 0.027 |
| Eyes Closed | ML | Both | Pre-Surgery | 2.270 | 0.032 | 0.038 |
| Eyes Closed | ML | Amputated | Pre-Surgery | 3.955 | 0.001 | 0.001 |
| Eyes Closed | ML | Intact | Pre-Surgery | -3.813 | 0.001 | 0.001 |
| Eyes Closed | AP | Both | Pre-Surgery | 3.802 | 0.001 | 0.001 |
| Eyes Closed | AP | Amputated | Pre-Surgery | 5.214 | 0.000 | 0.000 |
| Eyes Closed | AP | Intact | Pre-Surgery | -1.576 | 0.127 | 0.145 |
| Eyes Open | ML | Both | Post-Surgery | 2.309 | 0.029 | 0.037 |
| Eyes Open | ML | Amputated | Post-Surgery | 5.210 | 0.000 | 0.000 |
| Eyes Open | ML | Intact | Post-Surgery | -0.569 | 0.574 | 0.599 |
| Eyes Open | AP | Both | Post-Surgery | 6.744 | 0.000 | 0.000 |
| Eyes Open | AP | Amputated | Post-Surgery | 9.798 | 0.000 | 0.000 |
| Eyes Open | AP | Intact | Post-Surgery | -4.358 | 0.000 | 0.000 |
| Eyes Closed | ML | Both | Post-Surgery | 4.098 | 0.000 | 0.001 |
| Eyes Closed | ML | Amputated | Post-Surgery | 5.379 | 0.000 | 0.000 |
| Eyes Closed | ML | Intact | Post-Surgery | -0.608 | 0.549 | 0.599 |
| Eyes Closed | AP | Both | Post-Surgery | 5.033 | 0.000 | 0.000 |
| Eyes Closed | AP | Amputated | Post-Surgery | 6.303 | 0.000 | 0.000 |
| Eyes Closed | AP | Intact | Post-Surgery | -3.081 | 0.005 | 0.007 |
